# SNORA5A regulates tumor-associated macrophage M1/M2 phenotypes via *TRAF3IP3* in breast cancer

**DOI:** 10.1590/1414-431X2024e13809

**Published:** 2024-08-19

**Authors:** Yiqi Zhang, Ang Zheng, Yue Shi, Heng Lu

**Affiliations:** 1 Department of Surgery, The First Affiliated Hospital of Jinzhou Medical University, Jinzhou, China; 2 Department of Surgery, The First Affiliated Hospital of China Medical University, Shenyang, China

**Keywords:** SNORA5A, Breast cancer, Tumor-associated macrophage, TRAF3IP3, Immune microenvironment

## Abstract

Small nucleolar RNAs (snoRNAs) have robust potential functions and therapeutic value in breast cancer. Herein, we investigated the role SNORA5A in breast cancer. Samples from The Cancer Genome Atlas (TCGA) were reviewed. The transcription matrix and clinical information were analyzed using R software and validated in clinical tissue samples. SNORA5A was significantly down-regulated in breast cancer, and high expression of *SNORA5A* correlated with a favorable prognosis. High expression of SNORA5A induced a high concentration of tumor-associated macrophages M1 and a low concentration of tumor-associated macrophages M2. Moreover, SNORA5A were clustered in terms related to cancer and immune functions. Possible downstream molecules of SNORA5A were identified, among which TRAF3IP3 was positively correlated with M1 and negatively correlated with M2. The function of TRAF3IP3 in tumor inhibition and its relationship with macrophages in clinical tissue samples were in accordance with bioinformatics analysis results. SNORA5A could regulate macrophage phenotypes through *TRAF3IP3* and serves as a potential prognostic marker for breast cancer patients.

## Introduction

Breast cancer is the most common cancer and remains a significant threat to the health of women (1). Significant advances in medical practice related to breast cancer screening and therapy have been made, but innovative approaches are still critically needed to facilitate improvement in outcome and reduction in mortality (2). Management of multiple solid tumors has witnessed a new revolution with the advent of cancer immunotherapy and various immunotherapy modalities have been implemented in the fight against breast cancer (3,4). Thus, developing novel biomarkers that help to identify immune infiltration of tumor microenvironment and guide immunotherapy are areas of growing investigation (5).

Small nucleolar RNAs (snoRNAs), sized 60-300 bp, are a kind of non-coding RNA that originally function within the nucleolus and facilitate the modification of ribosomal RNAs (6,7). However, due to the assumption that snoRNAs activities were confined to the nucleolus, the significance of snoRNAs in cancer was largely ignored. Recently, the updated number and increasing evidence for snoRNAs have shown that the biological activities of snoRNA extends far beyond the nucleolus to the nucleus and even the cytoplasm (8). Accumulating studies show that abnormal regulation of snoRNAs contributes to cancer development, progression, and metastasis (9-12). In addition, it appears that snoRNAs are significant regulators in the immune system and have a role in the response to immunotherapies (13,14). SnoRNAs have robust potential as biomarkers and therapeutic targets in breast cancer.

Herein, we investigated the role SNORA5A in breast cancer. We investigated the expression of SNORA5A in breast cancer tissues and analyzed its relationship with various clinicopathological parameters. We used Gene set enrichment analysis (GSEA) to investigate *SNORA5A*-related signaling pathways. After that, we analyzed the possible role of SNORA5A in immune regulation and screened its regulated genes. *TRAF3IP3*, a SNORA5A up-regulated gene, is required for the development of T and B cells and highly expressed in immune organs and tissues. We analyzed its correlation with immune cells and related signaling pathways. Finally, the expression of TRAF3IP3 and its relation to macrophages were analyzed in breast cancer clinical specimens. Our data suggested that SNORA5A may be a tumor suppressor gene in breast cancer and affect the tumor microenvironment through *TRAF3IP3*, highlighting the potential role of SNORA5A in the breast cancer microenvironment, which has important clinical significance in terms of diagnosis and management for breast cancer patients.

## Material and Methods

### Breast cancer data collection

Breast cancer RNA-sequencing transcriptomic data and the corresponding clinical profiles were downloaded from The Cancer Genome Atlas (TCGA) program (https://portal.gdc.cancer.gov/). A total of 1,109 breast cancer samples and 113 normal breast tissue samples were obtained for the following analyses. After the completion of integration and standardization steps, breast cancer samples were divided into high and low expression groups according to the median value of the SNORA5A expression.

### Bioinformatics analysis

Immune scoring of breast cancer samples was performed, and samples were grouped according to the immune score level for differentially expressed snoRNAs analysis. Nine statistically significant differentially expressed snoRNAs were obtained, and among them SNORA5A was significantly upregulated in the group with high immune score (ESTIMATE R package and limma R package). Survival analysis was performed using the R survival package. Gene set enrichment analysis (GSEA) was performed to explore the enrichment pathways. The correlations between SNORA5A and tumor-infiltrating immune cells were calculated by CIBERSORT algorithm. We compared the expression data of the SNORA5A high expression samples with SNORA5A low expression samples using limma package of R (4.1.0) to identify the significant differentially expressed genes (DEG). Heatmaps and volcano plots were used to visualize the identified DEGs. Correlation analysis was performed between *SNORA5A* and the DEGs. The limma, ggExtra, reshape2, and ggpub packages based on R 4.1.0 were used for the analysis. Univariate Cox proportional hazard regression analysis was used to screen prognostic genes, with P<0.05 as the criteria. Correlation analysis was performed between 8 prognostic genes and immune cells, including TRAF3IP3, a gene highly associated with immune cells. The correlation between TRAF3IP3 and immune cells was calculated by the Pearson correlation using tidyverse, ggplot2, and ggExtra packages.

### Patients and tissue samples

The study was conducted in accordance with the Declaration of Helsinki and approved by the Ethical Committee of the JinZhou Medical University (approval No. 202289). A total of 118 breast cancer and 32 adjacent noncancerous paraffin-embedded specimens were obtained from the First Affiliated Hospital of JinZhou Medical University. Clinical and pathological information was obtained from the Hospital Information System, including age, tumor size, lymph node status, histologic grade, ER, PR, and HER2 indicators, and Ki-67 index.

### *In situ* hybridization (ISH)

ISH was performed using snoRNAs ISH Kit (Boster, China). The sections were firstly dewaxed by xylene and rehydrated by gradient ethanol. Then, endogenous peroxidase activity was blocked by 3% hydrogen peroxide, and mRNA was exposed by pepsin. Digoxin-labeled oligonucleotide probes were added in the hybridization solution and incubated overnight at 37°C. The next day, DAB staining was performed and SNORA5A expression was evaluated blindly by two pathologists. The intensity of positive cells was scored as 0 (negative), 1 (weak), 2 (moderate), and 3 (strong). The percentage of positive cells were scored as 0 (<5%), 1 (6-25%), 2 (26-50%), 3 (51-75%), and 4 (≥76%). The final scores were obtained by multiplying by intensity score and percentage score. Patients were categorized into two groups: high SNORA5A expression (score ≥4) and low SNORA5A expression (score <4).

### Immunohistochemistry (IHC)

IHC was performed using Ultra-sensitive™ S-P Kit (Maixin-Bio, China). Briefly, sections from paraffin-embedded tumor tissues were incubated with primary antibodies against TRAF3IP3 (1:200; Proteintech, China) and CD86 and CD206 (1:100; Proteintech) overnight at 4°C. Results were independently evaluated by two pathologists who were blinded to the experiment, as for ISH.

### Statistical analysis

All data were analyzed using R version 4.1.0 (R Core Team) and SPSS version 25.0 (IBM, USA). Survival curves were plotted by the Kaplan-Meier log rank test. The Pearson's chi-squared test was used to measure the correlation between protein expression and clinicopathological characteristic. Categorical variables are reported as counts and percentages. Two-tailed P<0.05 was considered statistically significant.

## Results

### Screening immune-related snoRNA, SNONRA5A

**Figure 1:**
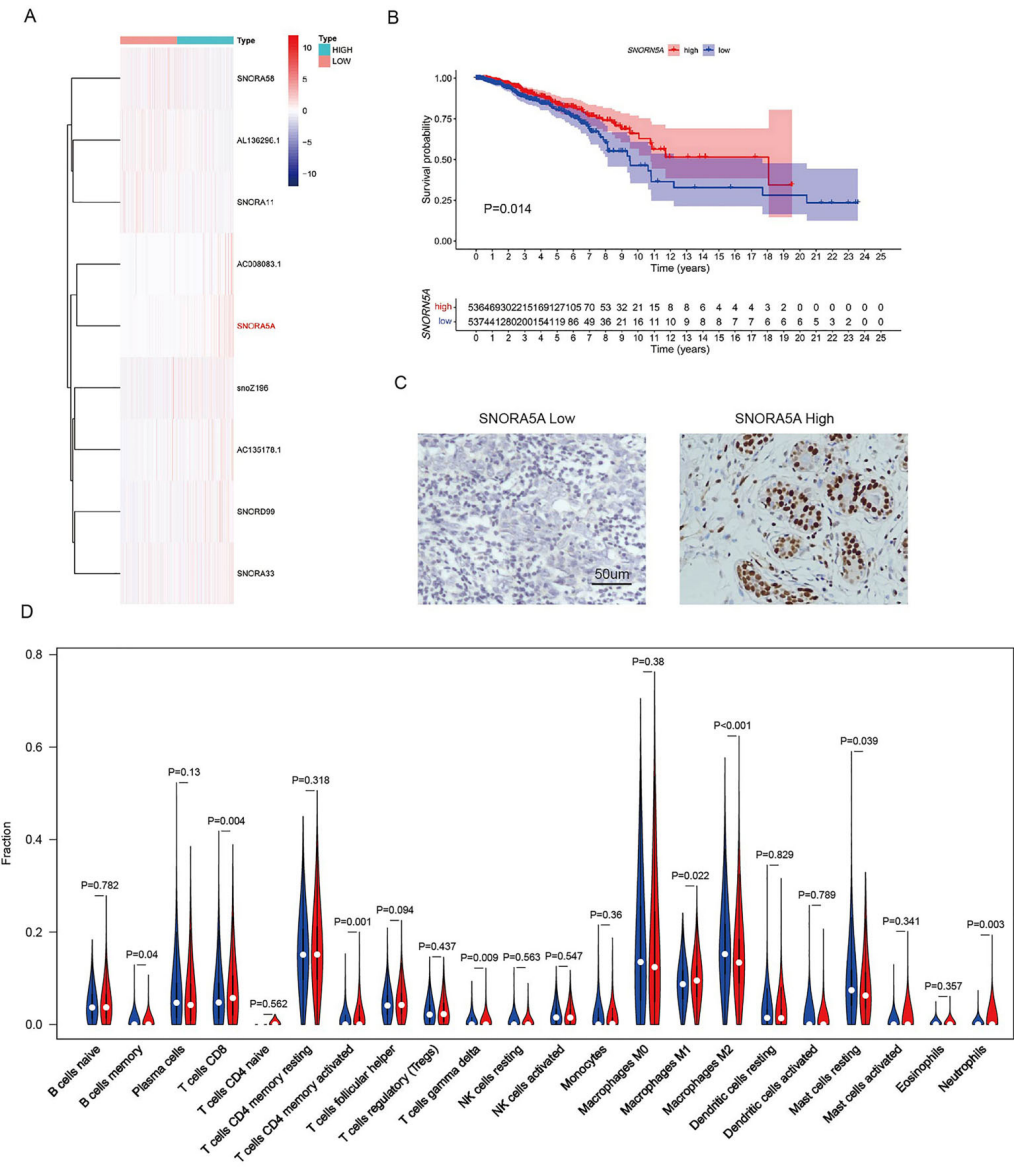
Screening of immune-related snoRNA in breast cancer. **A**, Differently expressed snoNRAs between the high and low immune score groups. **B**, Analysis of overall survival of groups divided by SNORA5A expression. **C**, *In situ* hybridization staining of SNORA5A expression (scale bar 50 μm). **D**, CIBERSORT results of immune cell infiltration differences between SNORA5A high (blue) and low (red) expression groups. The Wilcoxon test was used for statistical analysis.

To determine the differently expressed snoRNAs between the high and low immune score, we analyzed the transcriptome data from the TCGA database. As shown in the heatmap of Figure 1A, there were 9 differently expressed snoRNAs between the high and low immune score groups. SNORA5A was significantly up-regulated in the high immune core group. The survival analysis revealed that patients with high SNORA5A expression had a longer overall survival than those with low SNORA5A expression (Figure 1B). To further clarify the expression of SNORA5A in breast cancer, we evaluated SNORA5A expression levels in 118 breast cancer tissues and 32 normal breast tissues by ISH. The representative intensity for *SNORA5A* staining are shown Figure 1C. The positive rate (52/118, 44.1%) of *SNORA5A* in breast cancer tissues was significantly lower than that in adjacent normal breast tissues (21/32, 65.6%) (P<0.05). The association between SNORA5A expression and clinicopathological characteristics of the whole population are summarized in Table 1. The results showed that SNORA5A expression was significantly higher in T1-2 (P=0.017), N0 (P=0.012), and low Ki-67 index (P=0.009) group.

**Table 1:** Relationship between SNORA5A expression and clinicopathological features in breast cancer patients.

Factors	Number	SNORA5A High (%)	χ2	P
Total	118	52		
Age (years)			0.006	0.940
≤50	54	24 (44.4)		
>50	64	28 (43.8)		
T Stage			5.689	0.017*
T1-T2	87	44 (50.6)		
T3	31	8 (25.8)		
N Stage			6.353	0.012*
N0	76	40 (52.6)		
N1-3	42	12 (28.6)		
Histologic grade			0.480	0.489
G1-G2	99	45 (45.5)		
G3	19	7 (36.8)		
ER			0.213	0.644
Negative	25	10 (40.0)		
Positive	93	42 (45.2)		
PR			0.627	0.429
Negative	32	16 (50.0)		
Positive	86	36 (41.9)		
HER2			0.762	0.383
Negative	95	40 (42.1)		
Positive	23	12 (52.2)		
Ki-67 Index (%)			6.799	0.009*
≤20	73	39 (53.4)		
>20	45	13 (28.9)		

Data are reporter as numbers and percentages. *P<0.05 (chi-squared test). ER: estrogen receptor; PR: progesterone receptor.

To further analyze the relationship between SNORA5A and tumor immune cells, we calculated the invasion levels of immune cells in breast cancer tissues between SNORA5A high and low expression groups by the CIBERSORT algorithm. The proportion of 22 tumor-infiltrating immune cells in each subgroup are shown in Figure 1D. The results revealed that the SNORA5A high expression group had significantly higher proportions of B cell memory (P=0.04), CD8 T cells (P=0.004), T cell CD4 memory activated (P=0.001), T cells gamma delta (P=0.009), M1 macrophages (P=0.022), neutrophils (P=0.003), and a significantly lower proportion of M2 macrophages (P<0.001).

### SNORA5A-related signaling pathways

**Figure 2:**
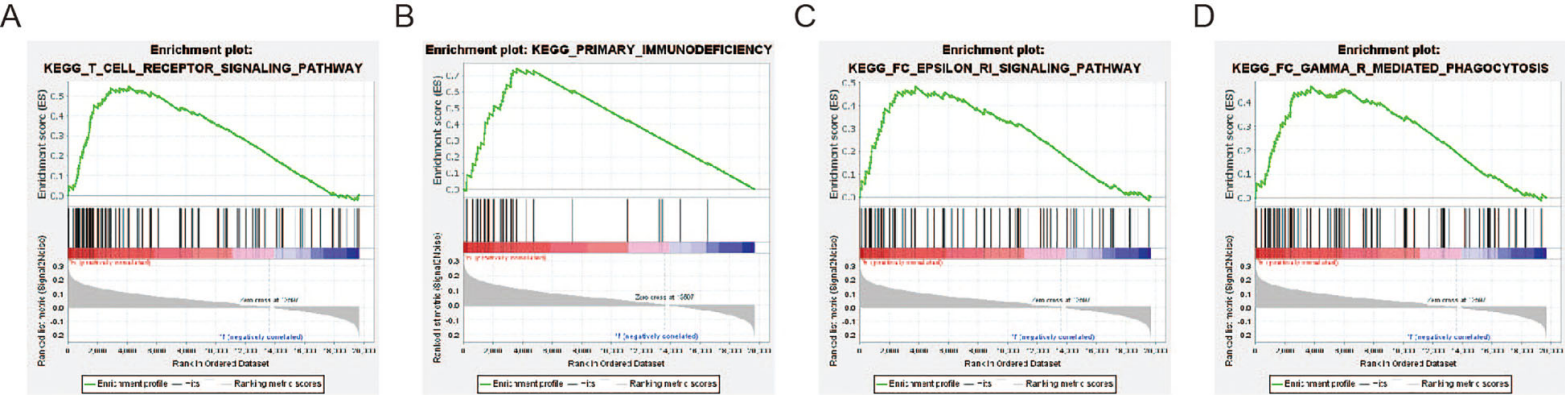
Gene set enrichment analysis (GSEA) of the mechanisms related to SNORA5A expression in breast cancer. GSEA disclosed a significant enrichment of (**A**) T cell receptor signaling pathway, (**B)** primary immunodeficiency, (**C**) fc epsilon ri signaling pathway, and (**D**) fc gamma r mediated phagocytosis.

To determine the identity of *SNORA5A*-related biological pathway, we chose significant enrichment of genes based on NES scores (Figure 2). The results indicated that *SNORA5A* expression was enriched in pathways of T cell receptor signaling pathway, primary immunodeficiency, fc epsilon ri signaling pathway, fc gamma r mediated phagocytosis, among others.

### Identification of SNORA5A-related prognostic genes

**Figure 3:**
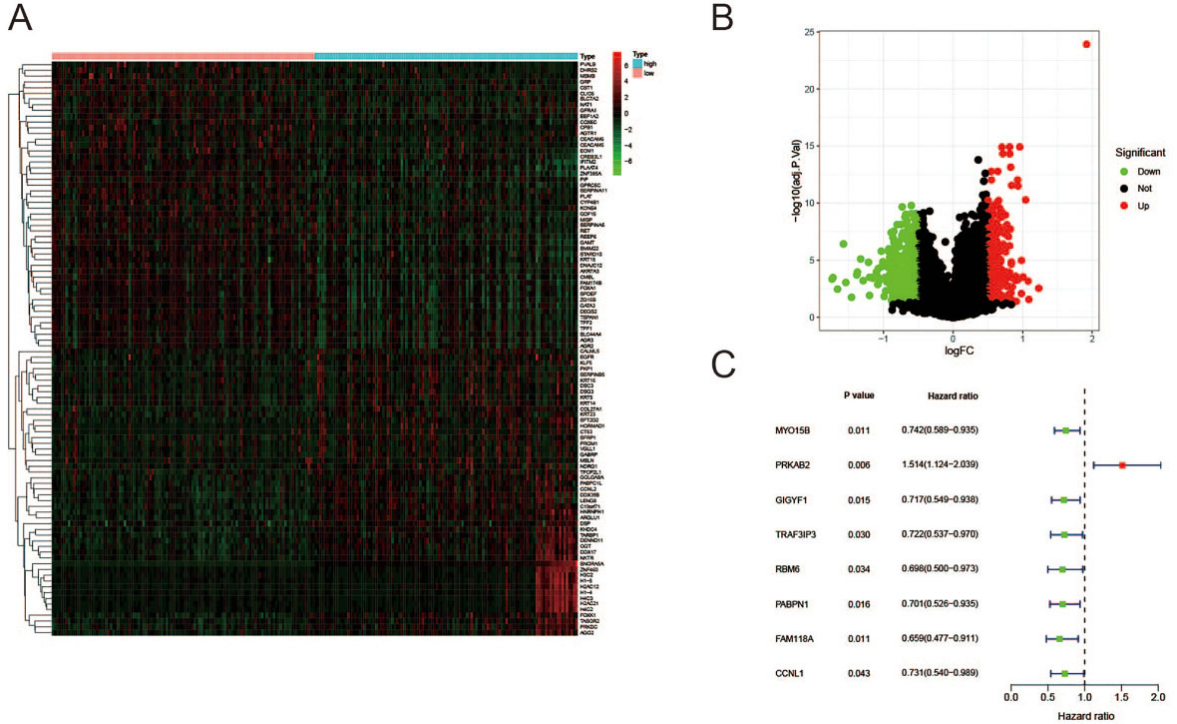
Identification of differentially expressed genes (DEGs) related to SNORA5A expression. **A**, Heatmap and (**B**) volcano plot of identified DEGs. **C**, Univariate Cox regression analyses of DEGs related to prognosis.

We analyzed the DEGs between *SNORA5A* high and low expression groups on the basis of |fold change|≥1 and adjusted P-value <0.05, as shown in the heatmap and volcano plot in Figure 3A and B. Among these DEGs, 281 genes were up-regulated and 416 genes were down-regulated. Thereafter, we subjected the DEGs to univariate Cox proportional hazard regression analyses, and 8 genes were identified as prognostic markers of overall survival in breast cancer patients (P<0.05) (Figure 3C). The correlation between *SNORA5A* and these 8 genes is shown in Supplementary Figure S1. Furthermore, the overall survival of patients was evaluated in accordance with the low or high expression of the 8 candidate genes. As shown in Supplementary Figure S2, a high expression of *MYO15B* (P=0.003), *GIGYF1* (P=0.009), *TRAF3IP3* (P=0.013), *RBM6* (P=0.025), *PABPN1* (P=0.014), *FAM118A* (P=0.036), and *CCNL1* (P=0.014) was associated with better prognosis, while a high expression of *PRKAB2* (P=0.003) was associated with poorer prognosis, suggesting that these 8 genes could be potential prognosis biomarkers for breast cancer patients.

### Analysis of TRAF3IP3 expression and its relationship with immune microenvironment

**Figure 4:**
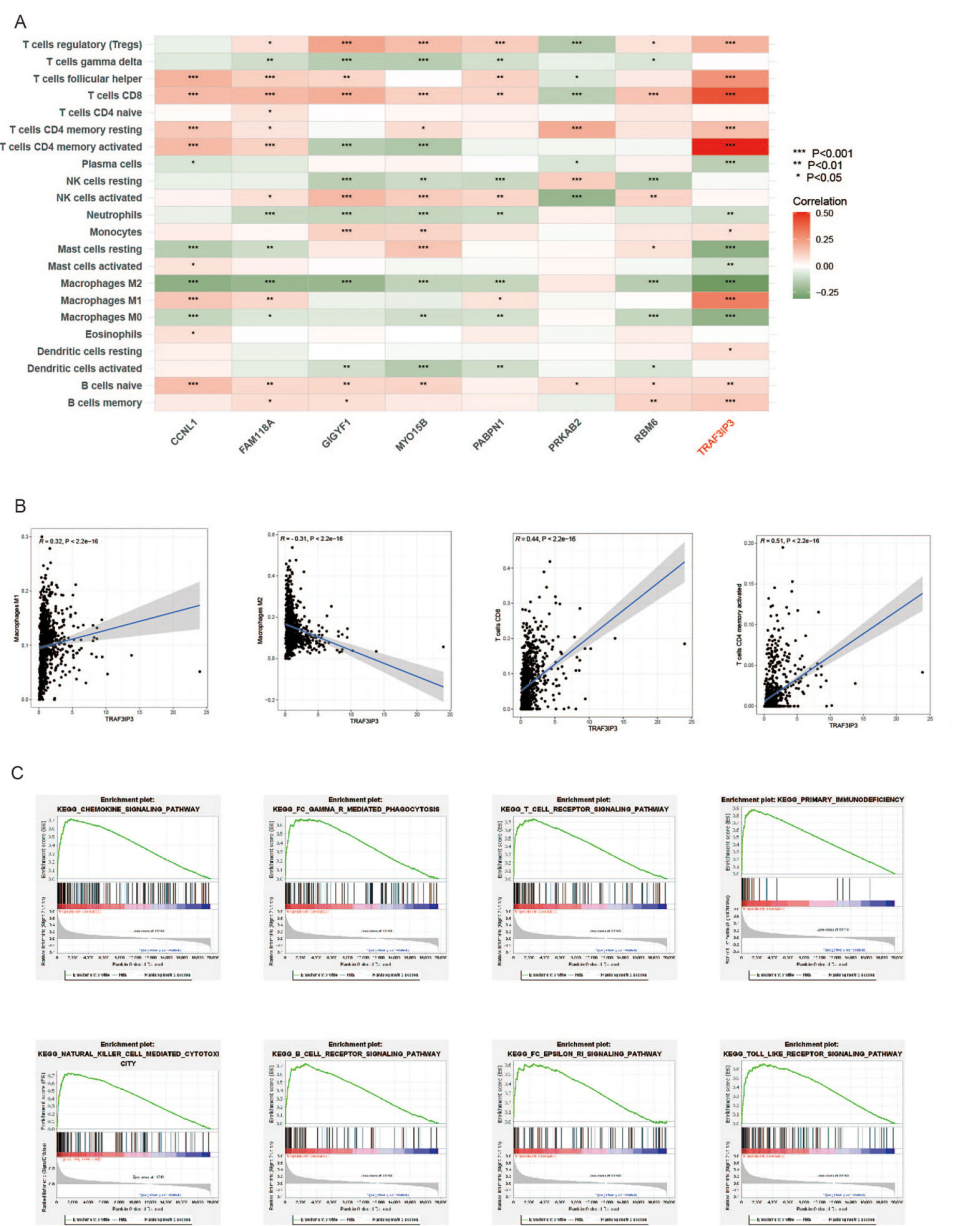
Analysis of relationships between TRAF3IP3 and immune microenvironment. **A**, Correlation of 8 candidate genes with immune cells. **B**, Correlation of TRAF3IP3 with CD8 T cells, T cell CD4 memory activated, macrophage M1, and macrophage M2. **C**, Gene set enrichment analysis of TRAF3IP3-related signaling pathways. The Signal2Noise was used for statistical analysis.

To gain insight into the effects of DEGs in the immune microenvironment, we investigated the relationship between 8 candidate genes and immune cells in breast cancer. Among them, TRAF3IP3 was accompanied with distinct infiltration status of various immune cells, including CD8 T cells, T cell CD4 memory resting, T cell CD4 memory activated, macrophage M1, macrophage M2, etc (Figure 4A). Consequently, correlation analyses confirmed that TRAF3IP3 positively correlated with CD8 T cells, T cell CD4 memory activated, and macrophage M1 and negatively correlated with macrophage M2 (P<0.001) (Figure 4B).

Subsequently, we explored the functions of *TRAF3IP3* in breast cancer by GSEA. *TRAF3IP3* mainly participated in terms related to immune functions such as chemokine signaling pathway, fc gamma r mediated phagocytosis, T cell receptor signaling pathway, primary immunodeficiency, natural killer cell mediated cytotoxicity, B cell receptor signaling pathway, fc epsilon ri signaling pathway, and toll-like receptor signaling pathway indicating that genes related by *TRAF3IP3* probably played an important role in immune-related processes in breast cancer (Figure 4C).

### Validation of TRAF3IP3 expression and its relationship with CD86 and CD206 in clinical tissue samples

The above results confirmed the correlation between the SNORA5A-related prognostic gene *TRAF3IP3* and macrophages in breast cancer. We proposed that the expression of SNORA5A may affect the tumor microenvironment through *TRAF3IP3*, and *TRAF3IP3* was chosen for further study. We evaluated the expression levels of TRAF3IP3 in 118 breast cancer tissues. The number of patients with high TRAF3IP3 expression was significantly higher in the T1-2 group (P=0.017) and N0 group (P=0.012) (Table 2).

**Table 2:** Relationship between TRAF3IP3 expression and clinicopathological features in breast cancer patients.

Factors	Number	TRAF3IP3 High (%)	χ2	P
Total	118	40		
Age (years)			0.014	0.905
≤50	54	18 (33.3)		
>50	64	22 (34.4)		
T Stage			3.969	0.046*
T1-T2	87	44 (50.6)		
T3	31	8 (25.8)		
N Stage			4.525	0.033*
N0	76	40 (52.6)		
N1-3	42	12 (28.6)		
Histologic grade			3.547	0.060
G1-G2	99	30 (30.3)		
G3	19	10 (52.6)		
ER			0.051	0.821
Negative	25	8 (32.0)		
Positive	93	32 (34.4)		
PR			0.254	0.614
Negative	32	12 (37.5)		
Positive	86	28 (32.6)		
HER2			0.153	0.696
Negative	95	33 (34.7)		
Positive	23	7 (30.4)		
Ki-67 Index (%)			0.815	0.367
≤20	73	27 (37.0)		
>20	45	13 (28.9)		

Data are reported as numbers and percentages. *P<0.05 (chi-squared test). ER: estrogen receptor; PR: progesterone receptor.

**Figure 5:**
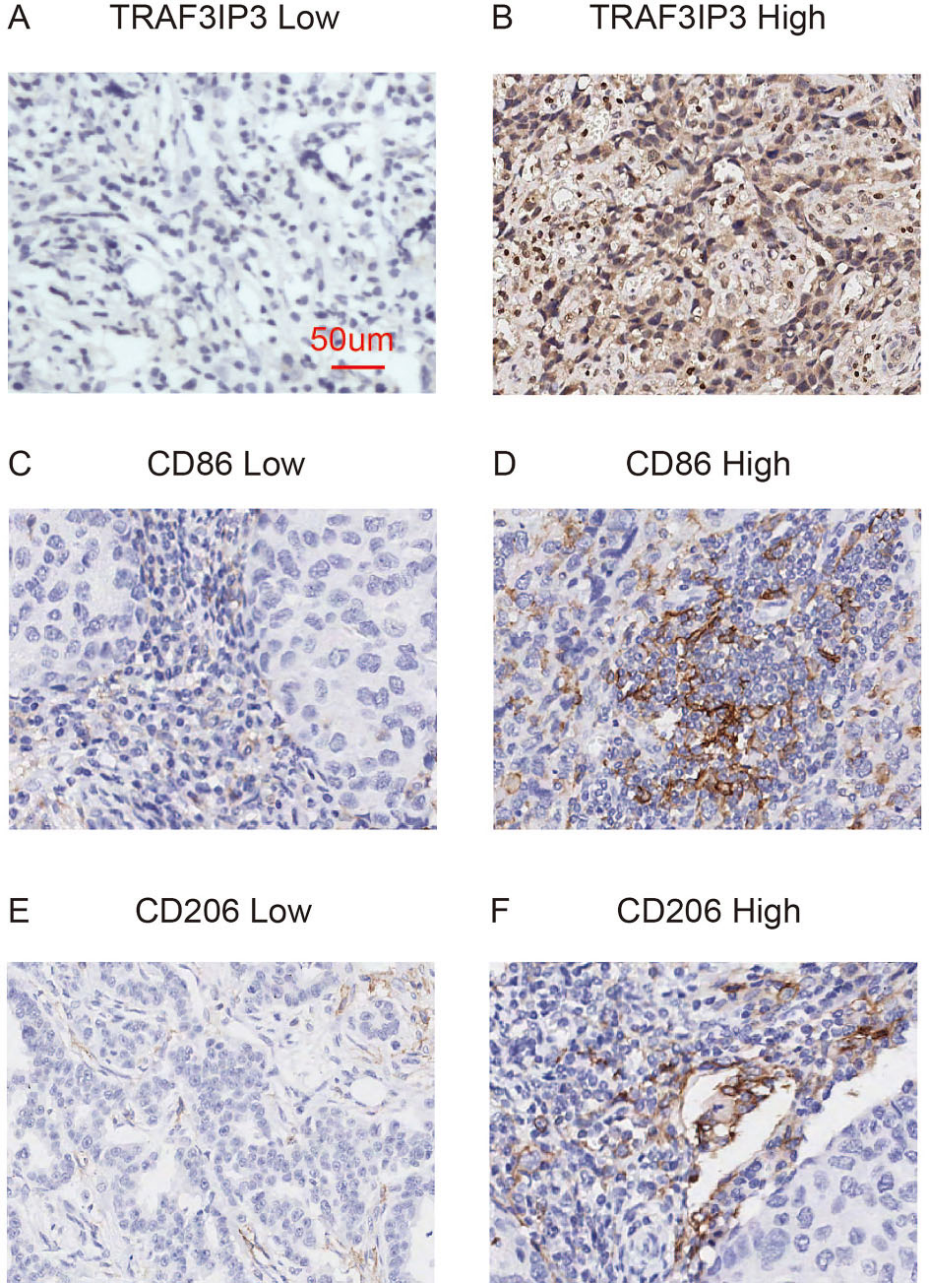
Immunohistochemical staining of TRAF3IP3, CD86, and CD206. **A** and **B**, Low and high expression of TRAF3IP3. **C** and **D**, Low and high expression of CD86. **E** and **F**, Low and high expression of CD206. Scale bar 50 μm.

CD86 is the M1-associated marker and CD206 is the M2-associated marker currently widely accepted (15). We selected anti-CD86 to evaluate M1 polarized macrophages and anti-CD206 to evaluate M2 polarized macrophages (Figure 5). The results showed that TRAF3IP3 high expression patients exhibited significantly higher staining of M1 macrophages (r=0.332, P<0.001), but lower staining of M2 macrophages (r=-0.212, P=0.020), which was consistent with bioinformatics analysis results (Table 3). In conclusion, TRAF3IP3 could influence tumor-associated macrophages and there was a more favorable immune environment in the TRAF3IP3 high group.

**Table 3:** Correlation analysis of TRAF3IP3 expression with CD86 and CD206.

Factors	TRAF3IP3 High (%)	TRAF3IP3 Low (%)	r	P
CD86 expression			0.332	<0.001
High (%)	25 (53.2)	22 (46.8)		
Low (%)	15 (21.1)	56 (78.9)		
CD206 expression			-0.212	0.020
High (%)	14 (23.0)	47 (77.0)		
Low (%)	26 (45.6)	31 (54.4)		

Pearson's correlation test was used for statistical analysis.

## Discussion

Breast cancer is the most common cancer in women. Immunotherapy is a new treatment modality that has created new opportunities for breast cancer patients. However, the use of immune checkpoint inhibitors has shown limited efficacy in patients with breast cancer (16). It is anticipated that by identifying specific immune-related biomarkers for detection and prediction the prognosis of breast cancer will be the way forward to improve the efficacy of immunotherapeutic approaches (17). Our study identified SNORA5A as a candidate for breast cancer suppressor and demonstrated its role in the immune microenvironment. These data may provide an alternate target for enhancing the immune response against breast tumors.

Recently, the potential contribution of snoRNAs in cancer immunity are gradually being revealed. SnoRNAs exhibit significant differential expression during normal and malignant hematopoiesis (13). An integrated multi-omics analysis including 823 advanced renal cell carcinoma patients revealed that patients in the snoRNA cluster exhibited the most favorable response to anti-PD-L1 (14). Tumor-infiltrating immune-related snoRNAs could be used to predict prognosis and responsiveness to immunotherapy in lung adenocarcinoma (18). However, the mechanisms by which snoRNA affects the microenvironment and prognosis of breast cancer is unclear, and elucidating the effects of new immune-related snoRNA related to breast cancer would be important. This study separated breast cancer patients into two groups based on the immune score and identified immune-related gene SNORA5A, providing a novel prognostic biomarker for breast cancer.

Generally, SNORA5A was down-regulated in advanced breast cancer patients, and patients in the SNORA5A high group had better overall survival. From GSEA, *SNORA5A* was enriched in several pathways including T cell receptor signaling pathway, primary immunodeficiency, fc epsilon ri signaling pathway, and fc gamma r mediated phagocytosis. These results indicated that SNORA5A exerts a critical role in breast cancer immune system processes. Most importantly, we found that the immune-infiltrating cells associated to SNORA5A were mainly macrophages (M1 and M2), indicating that SNORA5A has the most important influence on macrophages.

*TRAF3IP3*, as a SNORA5A-related prognostic gene, was first reported as a TRAF3 interacting protein (19). TRAF3IP3 is highly expressed in the immune system and is reportedly involved in B and T cell development (20,21). Later studies showed that TRAF3IP3 is essential for effective antiviral innate immune response by mediating TRAF3 recruitment to MAVS (22). Recent studies have confirmed the key role of TRAF3IP3 in tumor progression. High protein levels of TRAF3IP3 promote melanoma growth (23). In patients with glioma, TRAF3IP3 stimulates cell proliferation by activating the ERK signaling pathway, and high expression of TRAF3IP3 predicts a poor prognosis (24,25). In this study, we found that TRAF3IP3 expression was negatively associated with T stage and N stage of breast cancer patients. Furthermore, survival analyses suggested that patients had a better prognosis when they had a higher TRAF3IP3 expression. In our study, TARF3IP3 was involved in multiple immune-related signaling pathways. In accordance with a previous study (26), we found that TRAF3IP3 was significantly correlated with T cell. Furthermore, TARF3IP3 expression showed a positive correlation with M1 macrophage and a negative correlation with M2 macrophage.

TRAF3IP3 functions specifically in different subcellular localizations and alters protein localization. TRAF3IP3 mediates extracellular signal-regulated kinase signaling in the Golgi (21). TRAF3IP3 maintains the metabolic programs of regulatory T cell stability and function at the lysosome (26). In addition, TRAF3IP3 functions as an adapter molecule that interacts with TRAF3 to regulate its localization (19). A more recent study identified the nuclear localization signal and nuclear export signal of TRAF3IP3; the nuclear export signal contributes to the inhibition of EV71 and exhibits antiviral activity (27). Our hypothesis was that SNORA5A interacts with TRAF3IP3 and regulates its nuclear localization signal and nuclear export signal to exert its vital role in breast cancer immunity. Although our findings have improved the understanding of the relationship between SNORA5A and TRAF3IP3, which might provide a basis for future study, the underlying mechanisms still need to be further explored.

The tumor microenvironment is mainly composed of tumor cells, immune cells, mesenchymal cells, and extracellular matrix, among which macrophages are the key regulatory cell in breast cancer (28,29). Due to diverse functions and strong plasticity, macrophages efficiently respond to environmental signals and generally differentiate into classically activated M1 macrophages with tumoricidal effect or alternatively into activated M2 macrophages with tumor promoting effect (30,31). M1 macrophages are reprogrammed to transform to M2 macrophages upon specific stimuli, and M2 macrophages can be repolarized to M1 phenotype under certain conditions (32,33). Various studies have shown that tumor-associated macrophages are mainly polarized toward M2 macrophages in the tumor microenvironment and contribute to breast cancer progression through supporting tumor cell survival, angiogenesis, and metastasis (34-36). Therefore, it is particularly urgent to explore the mechanism of macrophage phenotype generation and transformation (37). Previous studies have found that the expression level of snoRNA changes significantly during macrophage polarization (38). We found that SNORA5A regulated the polarization of M1 and M2 macrophages through TRAF3IP3 in breast cancer. However, the molecular mechanism should be fully validated to support our study.

In summary, we explored an immune-related snoRNA in breast cancer, SNORA5A. SNORA5A serves as a potential diagnosis and prognosis marker of breast cancer. Moreover, we found that the infiltration levels of macrophages was significantly different among SNORA5A expression groups. Finally, a SNORA5A-related prognostic gene, *TRAF3IP3*, intensified M1 macrophage polarization and weakened M2 macrophage polarization. SNORA5A may affect macrophage polarization through *TRAF3IP3*. Collectively, SNORA5A may be a promising target for breast cancer immunotherapy.

## Supplementary Material

Click here to view [pdf].

## References

[1] Sung H, Ferlay J, Siegel RL, Laversanne M, Soerjomataram I, Jemal A (2021). Global Cancer Statistics 2020: GLOBOCAN estimates of incidence and mortality worldwide for 36 cancers in 185 countries. CA Cancer J Clin.

[2] Jacobs AT, Castaneda-Cruz DM, Rose MM, Connelly L (2022). Targeted therapy for breast cancer: an overview of drug classes and outcomes. Biochem Pharmacol.

[3] Emens LA, Ascierto PA, Darcy PK, Demaria S, Eggermont AMM, Redmond WL (2017). Cancer immunotherapy: opportunities and challenges in the rapidly evolving clinical landscape. Eur J Cancer.

[4] Barzaman K, Moradi-Kalbolandi S, Hosseinzadeh A, Kazemi MH, Khorramdelazad H, Safari E (2021). Breast cancer immunotherapy: current and novel approaches. Int Immunopharmacol.

[5] Huppert LA, Mariotti V, Chien AJ, Soliman HH (2022). Emerging immunotherapeutic strategies for the treatment of breast cancer. Breast Cancer Res Treat.

[6] Henras AK, Dez C, Henry Y (2004). RNA structure and function in C/D and H/ACA s(no)RNPs. Curr Opin Struct Biol.

[7] Lui L, Lowe T (2013). Small nucleolar RNAs and RNA-guided post-transcriptional modification. Essays Biochem.

[8] van der Werf J, Chin CV, Fleming NI (2021). SnoRNA in cancer progression, metastasis and immunotherapy response. Biology (Basel).

[9] Liao J, Yu L, Mei Y, Guarnera M, Shen J, Li R (2010). Small nucleolar RNA signatures as biomarkers for non-small-cell lung cancer. Mol Cancer.

[B10] Crea F, Quagliata L, Michael A, Liu HH, Frumento P, Azad AA (2016). Integrated analysis of the prostate cancer small-nucleolar transcriptome reveals SNORA55 as a driver of prostate cancer progression. Mol Oncol.

[B11] McMahon M, Contreras A, Holm M, Uechi T, Forester CM, Pang X (2019). A single H/ACA small nucleolar RNA mediates tumor suppression downstream of oncogenic RAS. Elife.

[12] Gong J, Li Y, Liu CJ, Xiang Y, Li C, Ye Y (2017). A pan-cancer analysis of the expression and clinical relevance of small nucleolar RNAs in human cancer. Cell Rep.

[13] Warner WA, Spencer DH, Trissal M, White BS, Helton N, Ley TJ (2018). Expression profiling of snoRNAs in normal hematopoiesis and AML. Blood Adv.

[14] Motzer RJ, Banchereau R, Hamidi H, Powles T, McDermott D, Atkins MB (2020). Molecular subsets in renal cancer determine outcome to checkpoint and angiogenesis blockade. Cancer Cell.

[15] Trombetta AC, Soldano S, Contini P, Tomatis V, Ruaro B, Paolino S (2018). A circulating cell population showing both M1 and M2 monocyte/macrophage surface markers characterizes systemic sclerosis patients with lung involvement. Respir Res.

[16] Esteva FJ, Hubbard-Lucey VM, Tang J, Pusztai L (2019). Immunotherapy and targeted therapy combinations in metastatic breast cancer. Lancet Oncol.

[17] Adams S, Gatti-Mays ME, Kalinsky K, Korde LA, Sharon E, Amiri-Kordestani L (2019). Current landscape of immunotherapy in breast cancer: a review. JAMA Oncol.

[18] Wan R, Bai L, Cai C, Ya W, Jiang J, Hu C (2021). Discovery of tumor immune infiltration-related snoRNAs for predicting tumor immune microenvironment status and prognosis in lung adenocarcinoma. Comput Struct Biotechnol J.

[19] Dadgostar H, Doyle SE, Shahangian A, Garcia DE, Cheng G (2003). T3JAM, a novel protein that specifically interacts with TRAF3 and promotes the activation of JNK (1). FEBS Lett.

[20] Peng S, Wang K, Gu Y, Chen Y, Nan X, Xing J (2015). TRAF3IP3, a novel autophagy up-regulated gene, is involved in marginal zone B lymphocyte development and survival. Clin Exp Immunol.

[21] Zou Q, Jin J, Xiao Y, Hu H, Zhou X, Jie Z (2015). T cell development involves TRAF3IP3-mediated ERK signaling in the Golgi. J Exp Med.

[22] Zhu W, Li J, Zhang R, Cai Y, Wang C, Qi S (2019). TRAF3IP3 mediates the recruitment of TRAF3 to MAVS for antiviral innate immunity. EMBO J.

[23] Nasarre P, Bonilla IV, Metcalf JS, Hilliard EG, Klauber-DeMore N (2018). TRAF3-interacting protein 3, a new oncotarget, promotes tumor growth in melanoma. Melanoma Res.

[24] Yang G, Tang S, Zhang J, Qin L (2021). High TRAF3IP3 level predicts poor prognosis of patients with gliomas. World Neurosurg.

[25] Lin Q, Chen Z, Shen ZL, Xue F, Qin JJ, Kang XP (2022). TRAF3IP3 promotes glioma progression through the ERK signaling pathway. Front Oncol.

[26] Yu X, Teng XL, Wang F, Zheng Y, Qu G, Zhou Y (2018). Metabolic control of regulatory T cell stability and function by TRAF3IP3 at the lysosome. J Exp Med.

[27] Li H, Yao Y, Chen Y, Zhang S, Deng Z, Qiao W (2022). TRAF3IP3 Is cleaved by EV71 3C protease and exhibits antiviral activity. Front Microbiol.

[28] Vitale I, Manic G, Coussens LM, Kroemer G, Galluzzi L (2019). Macrophages and metabolism in the tumor microenvironment. Cell Metab.

[29] Bahcecioglu G, Basara G, Ellis BW, Ren X, Zorlutuna P (2020). Breast cancer models: engineering the tumor microenvironment. Acta Biomater.

[30] Allavena P, Sica A, Solinas G, Porta C, Mantovani A (2008). The inflammatory micro-environment in tumor progression: the role of tumor-associated macrophages. Crit Rev Oncol Hematol.

[31] Martinez FO, Helming L, Gordon S (2009). Alternative activation of macrophages: an immunologic functional perspective. Annu Rev Immunol.

[32] Mehta AK, Kadel S, Townsend MG, Oliwa M, Guerriero JL (2021). Macrophage biology and mechanisms of immune suppression in breast cancer. Front Immunol.

[33] DeNardo DG, Ruffell B (2019). Macrophages as regulators of tumour immunity and immunotherapy. Nat Rev Immunol.

[34] Wu K, Lin K, Li X, Yuan X, Xu P, Ni P (2020). Redefining tumor-associated macrophage subpopulations and functions in the tumor microenvironment. Front Immunol.

[B35] Su S, Liu Q, Chen J, Chen J, Chen F, He C (2014). A positive feedback loop between mesenchymal-like cancer cells and macrophages is essential to breast cancer metastasis. Cancer Cell.

[36] Alhudaithi SS, Almuqbil RM, Zhang H, Bielski ER, Du W, Sunbul FS (2020). Local targeting of lung-tumor-associated macrophages with pulmonary delivery of a CSF-1R inhibitor for the treatment of breast cancer lung metastases. Mol Pharm.

[37] Choi J, Gyamfi J, Jang H, Koo JS (2018). The role of tumor-associated macrophage in breast cancer biology. Histol Histopathol.

[38] Ma D, Zhou X, Wang Y, Dai L, Yuan J, Peng J (2022). Changes in the small noncoding RNAome during M1 and M2 macrophage polarization. Front Immunol.

