# Supplementary material for: SNORA5A regulates tumor-associated macrophage M1/M2 phenotypes via TRAF3IP3 in breast cancer
Source: Braz J Med Biol Res. 2024 Aug 19;57:e13809. doi: 10.1590/1414-431X2024e13809 (PMC11338549; doi:10.1590/1414-431X2024e13809)

Figure S1. Correlation analysis between SNORA5A and SNORA5A-related prognostic genes, including (**A**) *MYO15B*, (**B**) *PPKAB2*, (**C**) *GIGYF1*, (**D**) *TRAF3IP3*, (**E**) *RBM6*, (**F**) *PABPN1*, (**G**) *FAM118A*, and (**H**) *CCNL1*. Pearson's correlation test was used for statistical analysis.

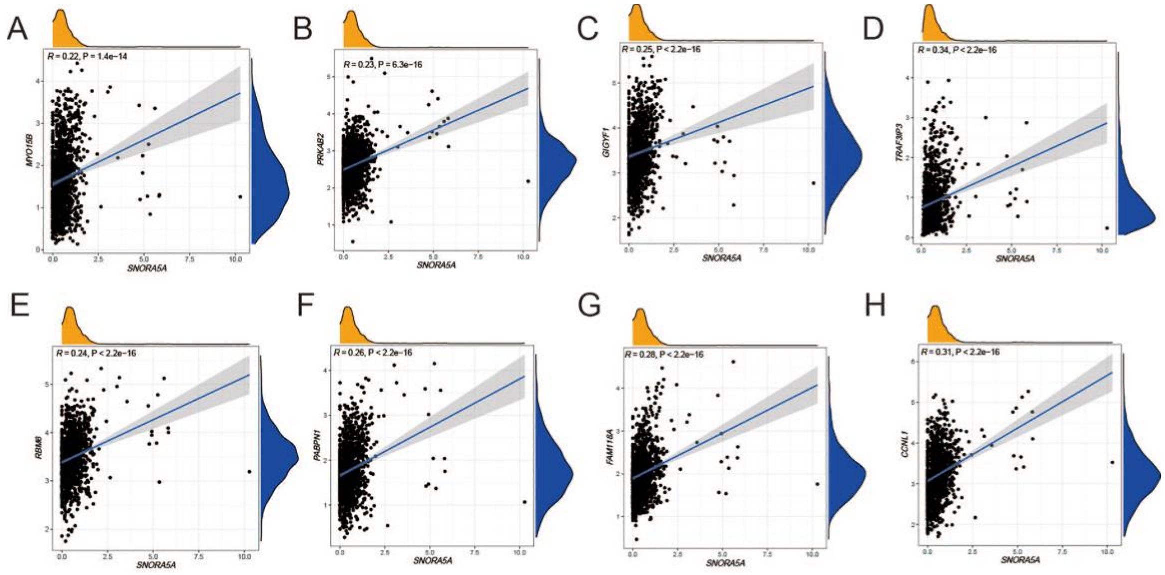

Figure S2. Survival analysis of SNORA5A-related prognostic genes, including (**A**) *MYO15B*, (**B**) *PPKAB2*, (**C**) *GIGYF1*, (**D**) *TRAF3IP3*, (**E**) *RBM6*, (**F**) *PABPN1*, (**G**) *FAM118A*, and (**H**) *CCNL1*. Cox analysis was used for statistical analysis.

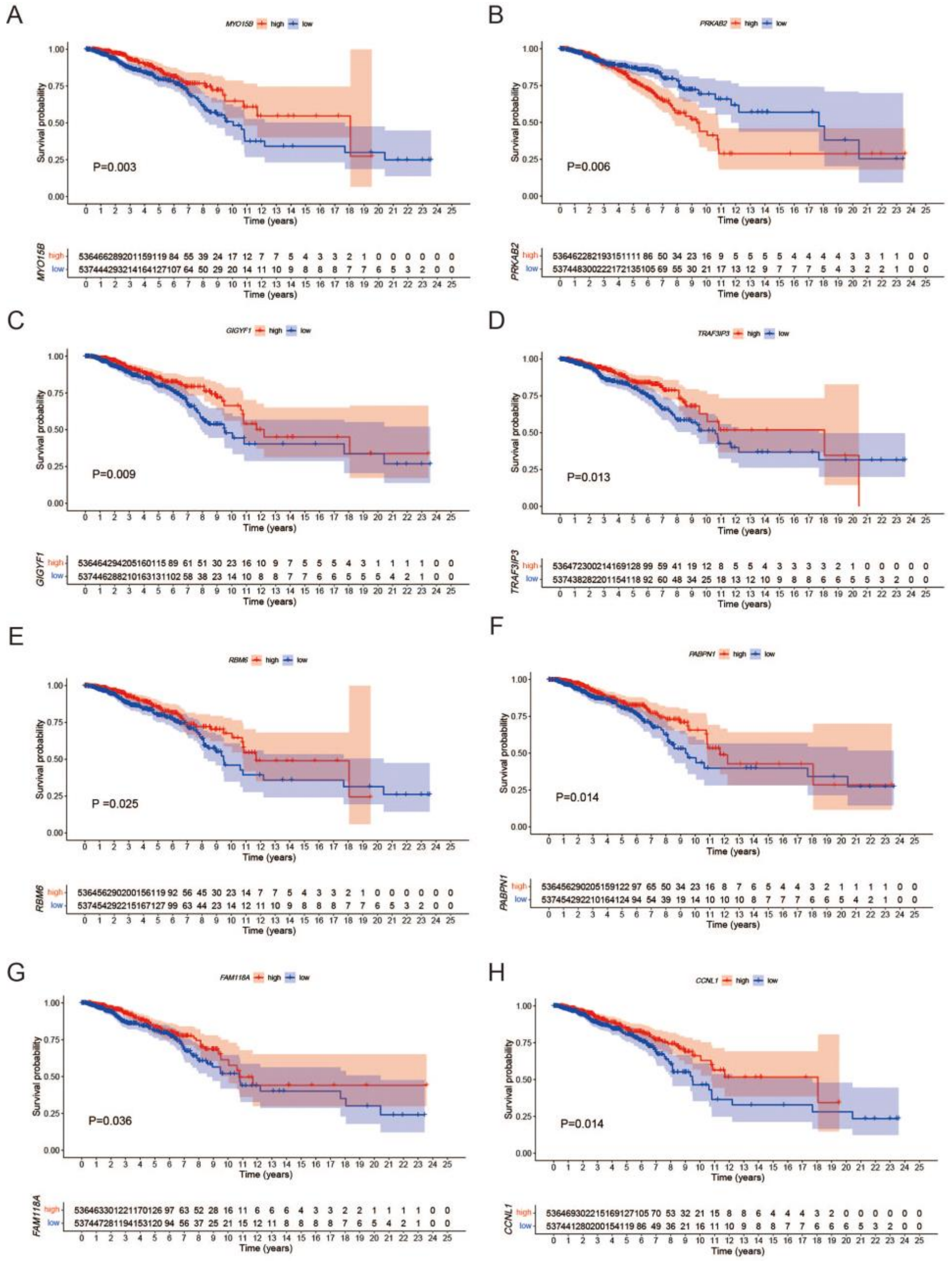

Supplement: Supplementary file 1 [file 1414-431X-bjmbr-57-e13809-suppl.pdf]
